# Vogt-Koyanagi-Harada disease is always bilateral: reports of unilateral cases failed to use choroidal investigations showing subclinical involvement of the fellow eye

**DOI:** 10.1186/s12348-021-00237-3

**Published:** 2021-02-09

**Authors:** Ioannis Papasavvas, Carl P. Herbort

**Affiliations:** Retinal and Inflammatory Eye Diseases, Centre for Ophthalmic Specialized Care (COS), Teaching Centre Clinic Montchoisi, Lausanne, Switzerland

**Keywords:** Vogt-Koyanagi-Harada disease, Indocyanine green angiography, Autoimmune stromal choroiditis

## Abstract

**Background/purpose:**

Vogt-Koyanagi-Harada (VKH) disease is a primary stromal choroiditis and a bilateral granulomatous panuveitis which, if not treated early and properly, could have a deleterious evolution. The purpose of our case report is to demonstrate that “so called” unilateral VKH disease should be investigated further with an Indocyanine Green Angiography (ICGA), in order to detect subclinical choroidal involvement of the other eye.

**Case report:**

We present a case of 42-year old woman who came to see us for second opinion. She had consulted elsewhere for a left uveitis and had been treated with a periocular corticosteroid injection. At presentation she mentioned persistent headaches. Visual acuity on the Snellen scale was 1.0 OD and 0.5 OS. Slit-lamp examination showed granulomatous (rare mutton-fat KPs) signs in her left eye. Laser flare photometry showed a subclinical flare of 17.8 ph/ms OD and a flare of 66.4 ph/ms OS (normal values 3–6 ph/ms). Fundus examination showed left discoloration due to choroidal infiltration with a normal fundus aspect OD. ICGA showed a diffuse choroiditis also in the apparently normal right eye. Lumbar puncture confirmed the diagnosis of VKH and appropriate treatment was introduced.

**Conclusion:**

VKH disease results from a generalized autoimmune process against melanocyte associated antigens starting in the choroidal stroma. It can be asymmetrical but is always bilateral, as long as investigations such as ICGA, able to detect subclinical choroiditis, are performed.

## Introduction

Vogt-Koyanagi-Harada (VKH) disease is a primary stromal choroiditis caused by an autoimmune reaction against melanocyte associated proteins [1]. Ocular disease is associated with acute systemic manifestations including inflammation of the meninges (CSF mononuclear pleiocytosis) and auditory disturbances and later, if the disease is left to evolve, with integumentary changes (vitiligo, alopecia and poliosis). This is the way VKH is classically described in articles and textbooks and corresponds, actually to non-treated or undertreated disease evolving from acute to chronic disease [2]. The first comprehensive series was reported by Koyanagi in 1929, describing the natural course of the disease at a time when no treatment was available, not altering the natural course of the 16 reported cases which were all bilateral [3]. Likewise, in the report by Harada in 1926 on 5 cases with posterior findings of what is now called VKH disease, the involvement was bilateral [4]. This is how he described the time course: “The patients developed onset of visual disturbance in both eyes, either simultaneously or with a few days of delay from one eye to the other” [4].

It is important to be aware of the disease mechanism at the origin of VKH disease, namely a primary stromal choroiditis, generated by an autoimmune reaction against proteins associated with stromal melanocytes [1, 5]. This means that inflammation exclusively starts within the choroidal stroma and only secondarily spills over to involve the other compartments of the eye [6]. Until the mid-1950s no inflammation suppressive treatment was available. Since the mid-1950s corticosteroids started to be given, however usually with delay, as it was still an experimental treatment [7, 8]]. This explains why the reported cases until effective treatment became available, were always bilateral [9–11]. Indeed, lack of efficient management allowed asymmetrical cases to become bilateral.

“Unilateral” cases started to be described since efficient inflammation suppressive therapy such as corticosteroid therapy or other became available and was used early in the disease. In these cases, the typical clinical presentation in one eye led to prompt systemic treatment so that the subclinical involvement of the contralateral eye was suppressed and never emerged. Since the mid-1990s, precise investigation of choroidal inflammation has become possible thanks to ICGA, allowing to detect subclinical disease during initial onset disease and to monitor occult subclinical recurrence during follow-up. To a lesser extent this is also possible with Enhanced Depth Imaging Optical Coherence Tomography (EDI-OCT) [12–14].

At least 11 cases of “unilateral” VKH disease in 8 articles were found in the literature (Table 1) [15–22]. None of them performed the investigations capable to show subclinical inflammatory involvement of the choroid in the other eye such as ICGA or EDI-OCT. [23, 24] We report a case of “so-called unilateral” VKH disease that in reality presented bilateral involvement, which was clinically apparent in one eye and subclinical in the contralateral eye with lesions only detected by ICGA.

**Table 1:** List of “unilateral” VKH cases

First author	Journal	Ref	Year	Number of cases
Kouda N et al.	Jpn J Ophthalmol	[12]	2002	1 case
Usui Y et al.	Graefes Arch Clin Exp Ophthalmol	[13]	2009	3 cases
Yokoi R et al.	Retin Cases Brief Rep	[14]	2010	1 case
Agrawal A & Biswas J	Middle East Afr J Ophthalmol	[15]	2011	2 cases
Neves A et al.	Case Rep Ophthalmol	[16]	2015	1 case
Valenzuela RM et al.	Neuro-Ophthalmology	[17]	2016	1 case
Tsui E et al.	Ocul Immunol Inflamm	[18]	2018	1 case
Pellegrini F et al.	Neuro-Ophthalmology	[19]	2018^a^	1 case
Hosseini SM et al.	J Ophthalmic Vis Res	[24]	2020^a^	1 case
				Total: 12 cases

^a^ Reports in which VKH was termed as unilateral although ICGA was performed and showed bilaterality

## Case report

A 42-year old woman consulted the Centre for Ophthalmic Specialised Care (COS) because of decreased visual acuity in her left eye. She had been seen several weeks earlier for photopsias. She had been investigated for uveitis but, allegedly, investigations were negative and a periocular corticosteroid injection had been administered on the left side.

**Fig. 1:**
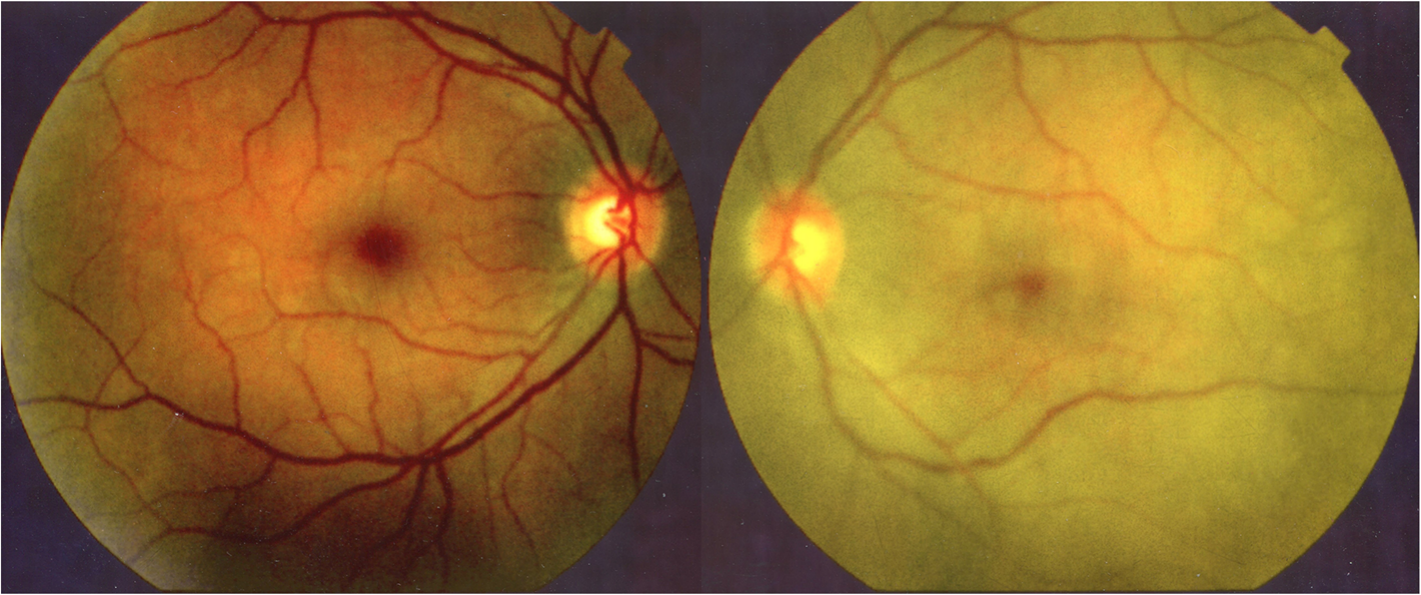
Case of VKH at presentation. Fundus picture showing yellow discoloration of the left eye due to choroidal infiltration while the right fundus is normal

When we saw the patient, she complained of persistent headaches and her visual acuity on the Snellen scale was 1.0 OD and 0.5 OS. At the slit-lamp there was no flare OD and a 1+ flare OS. Laser flare photometry showed a subclinical flare of 17.8 ph/ms OD and a flare of 66.4 ph/ms OS (normal values 3–6 ph/ms). On the left there were rare mutton-fat keratic precipitates and a few Koeppe nodules as well as 1+ of anterior vitreous cells. Fundus examination showed left yellow discoloration indicating choroidal infiltration while the right fundus was normal (Fig. 1).

**Fig. 2:**
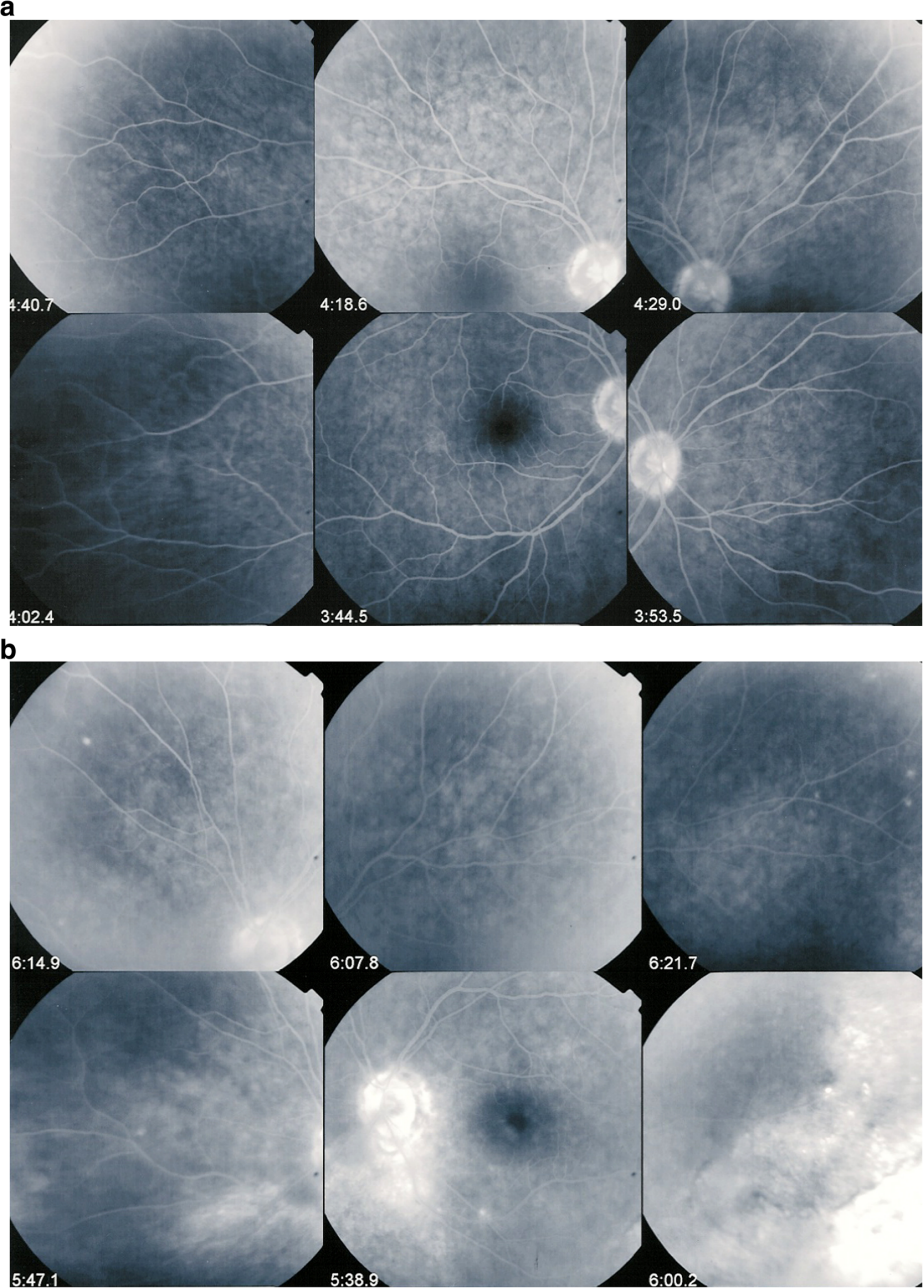
**a** FA OD of VKH case at presentation. FA is within normal limits. **b** FA OS of VKH case at presentation. FA shows disc hyperfluorescent and hyperfluorescent areas temporo-inferiorly

**Fig. 3:**
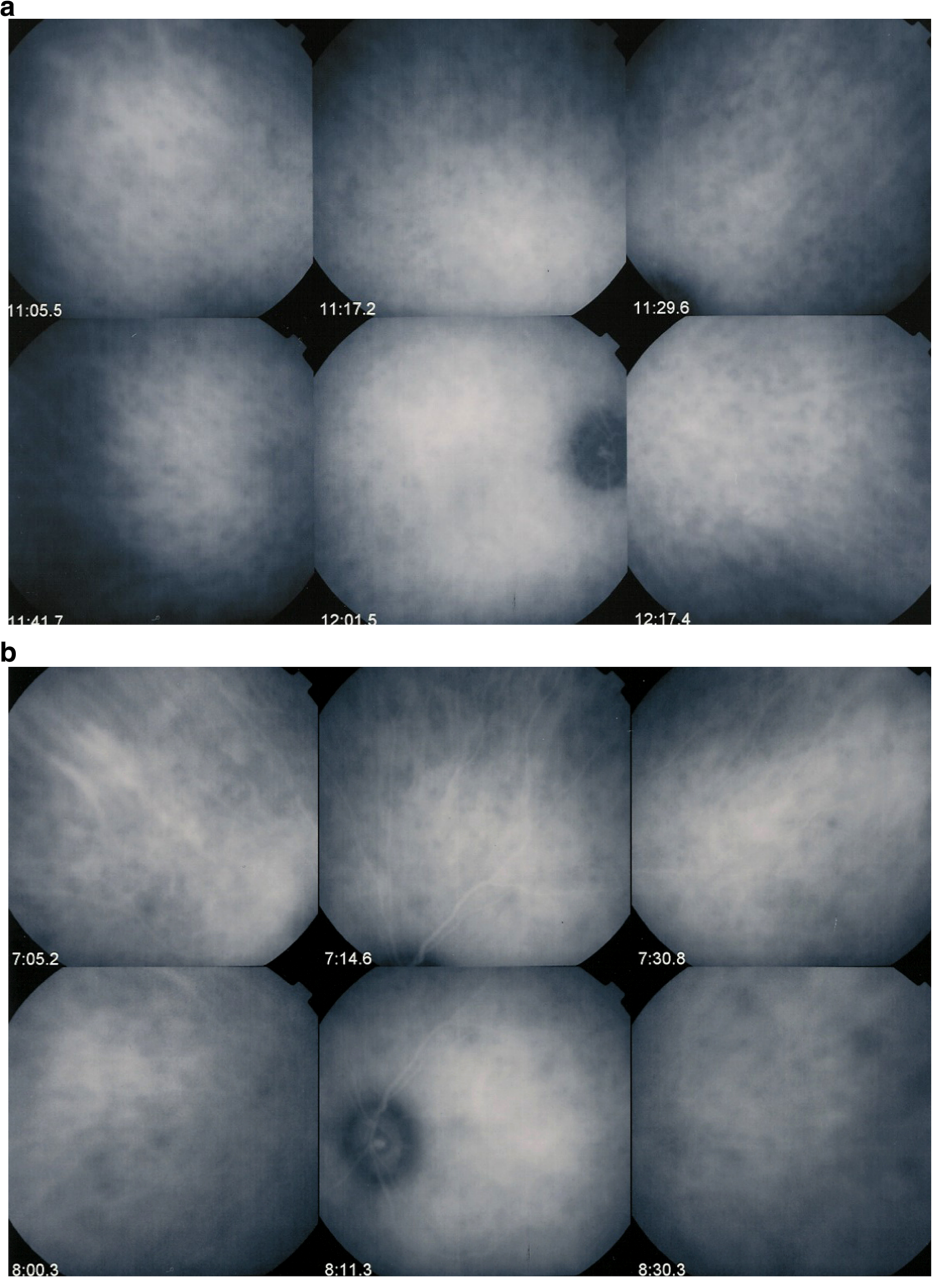
**a** ICGA OD of VKH case at presentation. ICGA shows numerous evenly distributed HDDs and loss of identification of choroidal vessels, while fundus picture and FA are normal, identifying subclinical diffuse choroiditis. **b** ICGA OS of VKH case at presentation. ICGA shows numerous evenly distributed HDDs and loss of identification of choroidal vessels and ICGA disc hyperfluorescence indicating sever inflammation

Fluorescein angiography was normal in the right eye with the exception of a slightly hyperfluorescent disc, while on the right eye there was a pronounced disc hyperfluorescence and several areas of hyperfluorescence inferior-temporally (Fig. 2a & b). ICGA, however, showed bilateral hypofluorescent dark dots (HDDs) on both sides indicating bilateral diffuse choroiditis (Fig. 3a & b).

**Fig. 4:**
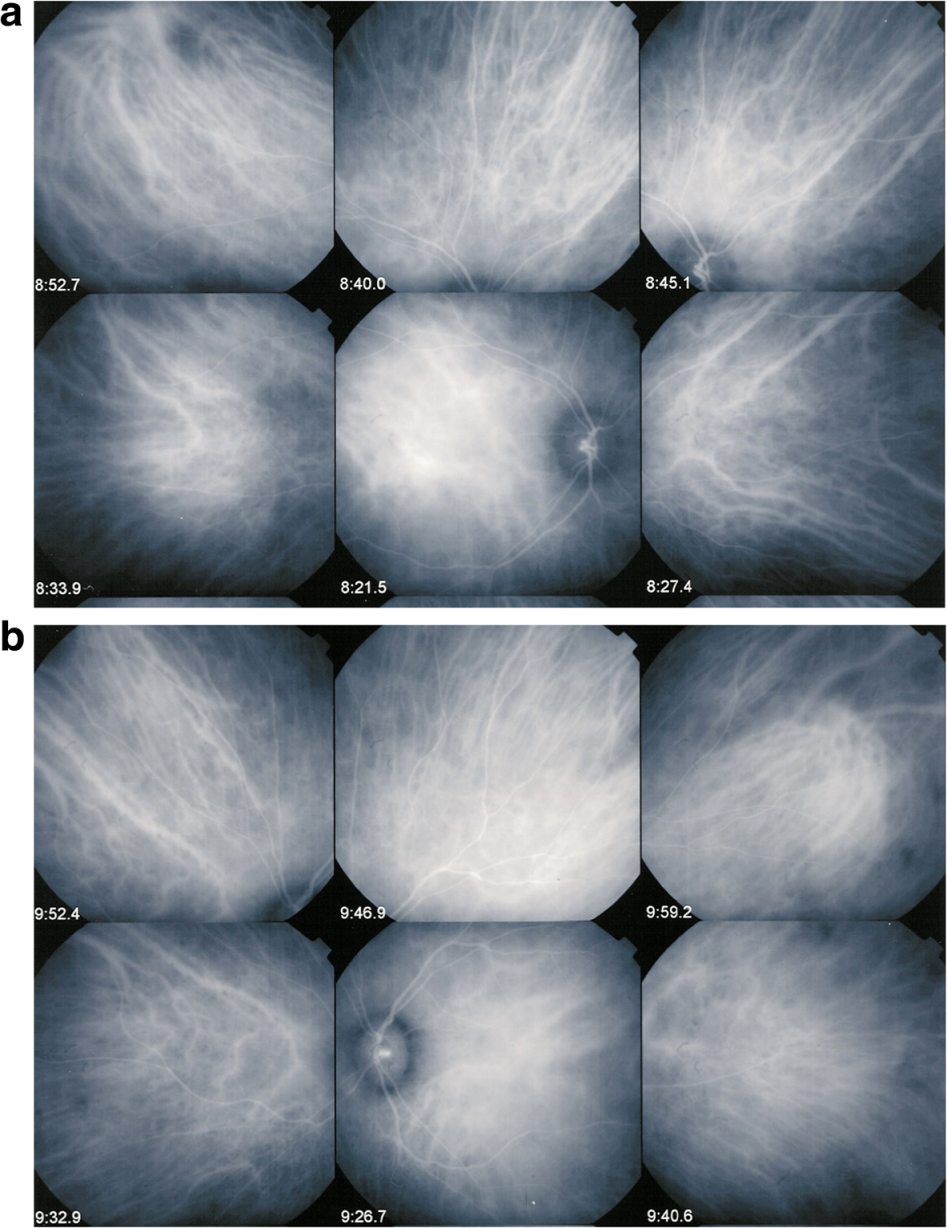
Fundi of VKH case 2 months after dual steroidal and non-steroidal immunosuppression. The left fundus shows again a normal colour

**Fig. 5:**
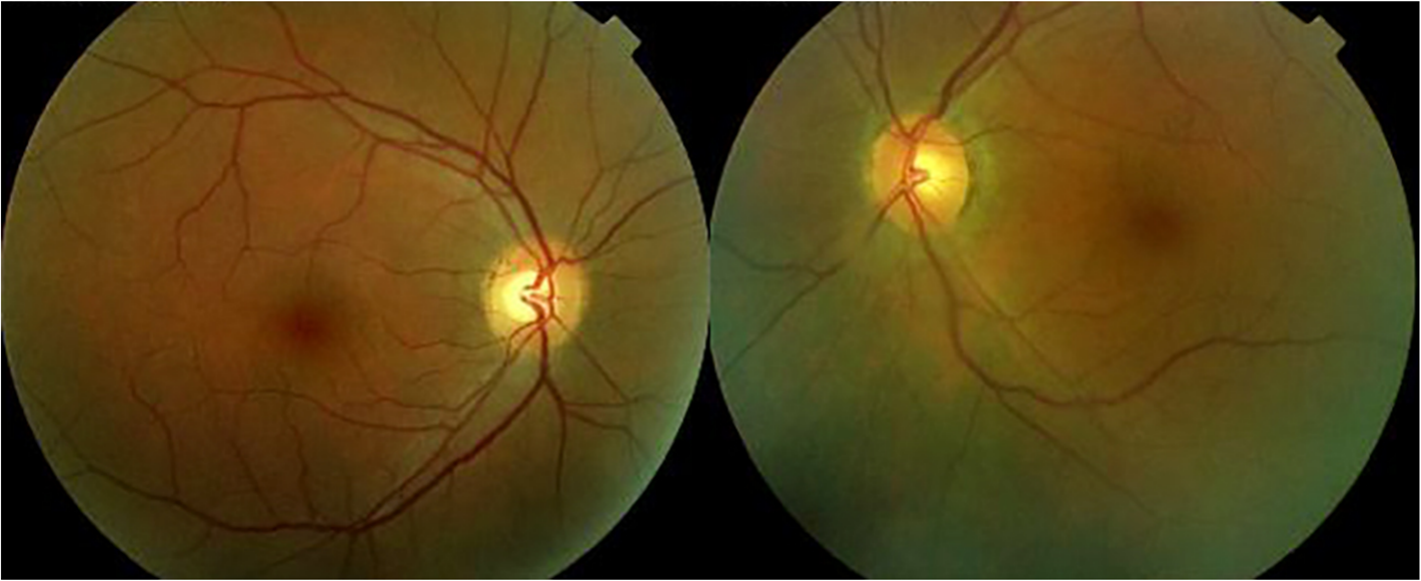
**a** ICGA OD of VKH case 2 months after dual steroidal and non-steroidal immunosuppression. ICGA shows disappearance of HDDs and clear visualisation of normalized choroidal vessels. **b** ICGA OS of VKH case 2 months after dual steroidal and non-steroidal immunosuppression. ICGA shows vanishing of HDDs and again clear visualisation of normal-appearing choroidal vessels

Anticipating vigorous dual steroidal and non-steroidal immunosuppression, in order to be sure of the diagnosis and to have the patient accept the therapy we performed a lumbar puncture showing a pleiocytosis of 243/3 lympho-monocytes in the cerebrospinal fluid. Dual corticosteroid and cyclosporin therapy was initiated at once. Two months later, under therapy, the colour of the fundus OS returned to normal (Fig. 4a & b), FA showed decrease of hyperfluorescence of the optic disc and temporal inferior area of hyperfluorescence and ICGA showed disappearance of HDDs and restoration of the choroidal vessels (Fig. 5a & b). No optical coherence tomography (OCT) data were available as the patient was seen in the pre-SD-OCT and pre-EDI-OCT era.

No clinically apparent disease developed on the right side. Unfortunately, after an attempt to taper therapy one year later, the disease recurred bilaterally and the patient had to be treated during the following 18 years with diverse combinations of inflammation suppressive therapies including anti-TNF agents.

## Discussion

VKH disease results from a generalized autoimmune aggression of melanocyte related proteins starting in the choroidal stroma and extending anteriorly in the eye and to other sites if treatment is not introduced without delay [6]. Asymmetrical ocular involvement can happen and has been described, starting with Harada’s report [4]. In the ground-breaking report of Koyanagi in 1929, two of the 4 new cases reported had a delayed involvement of the second eye [3]. Lavezzo and coll. Indicate that there is a delay of involvement of the second eye in 30% of cases [25]. In the absence of inflammation suppressive therapy before the mid-1950s or thereafter, when corticosteroid therapy was usually applied with delay [7, 8], ultimately all cases developed bilaterality, even those cases with initially unilateral presentation. Introduction of rapid and efficient therapy was at the origin of the reports on apparently unilateral cases [26]. Indeed, prompt treatment following clinically apparent disease in one eye resolved uveitis in the affected eye but also suppressed the subclinical disease in the contralateral eye. The latter eye therefore never developed clinically apparent disease. In the “unilateral” cases reported, two patients were investigated by ICGA that showed bilateral ICGA signs, one of which showed discreet signs in the apparently uninvolved eye before full-blown ICGA signs appeared 2 months later [27]. An interesting report showed delayed involvement of the second eye after the first eye showed VKH signs [28]. Unfortunately no OCT data were available as the case was seen in the pre-EDI-OCT era. It would be interesting to find out whether this imaging modality would be able to uncover subclinical involvement in the fellow eye. Subsequent evolution of the cases was available in two articles. In the first one sunset glow fundus (SGF) developed in both eyes confirming involvement of the fellow eye [12]. In the 3 cases of the second article, the fellow eyes did not develop SGF, probably indicating that efficient therapy was introduced early enough to prevent depigmentation in the contralateral eye [13].

## Conclusion

In essence there is no such thing as unilateral VKH. All those VKH cases that were labelled as unilateral only because proper investigations to detect choroidal subclinical inflammation in the other eye were not performed.

## Data Availability

The data used during the current article are available from the corresponding author on reasonable request.

## Abbreviations

VKH: Vogt-Koyanagi-Harada
ICGA: Indocyanine Green Angiography
CSF: Cerebrospinal Fluid
HDDs: Hypofluorescent dark dots
EDI-OCT: Enhanced Depth Imaging Optical Coherence Tomography
